# The influence of gender dynamics on polio eradication efforts at the community, workplace, and organizational level

**DOI:** 10.1186/s41256-021-00203-5

**Published:** 2021-06-29

**Authors:** Anna Kalbarczyk, Aditi Rao, Adedamola Adebayo, Ellie Decker, Sue Gerber, Rosemary Morgan

**Affiliations:** 1 Department of International Health, Johns Hopkins Bloomberg School of Public Health, 415 N Wolfe St, Baltimore, MD USA; 2 University of Ibadan College of Medicine, Ibadan, Nigeria; 3 Bill and Melinda Gates Foundation, Seattle, USA

**Keywords:** Polio eradication, Gender, Leadership, Health workforce

## Abstract

**Background:**

Globally, gender as a barrier or facilitator in achieving health outcomes is increasingly being documented. However, the role of gender in health programming and organization is frequently ignored. The Global Polio Eradication Initiative, one of the largest globally coordinated public health programs in history, has faced and worked to address gender-based challenges as they emerge. This paper seeks to describe the role of gender power relations in the polio program across global, national, subnational, and front-line levels to offer lessons learned for global programs.

**Methods:**

We conducted qualitative key-informant interviews with individuals purposively selected from the polio universe globally and within seven country partners: Afghanistan, Bangladesh, the Democratic Republic of the Congo, Ethiopia, India, Indonesia, and Nigeria. The interview tool was designed to explore nuances of implementation challenges, strategies, and consequences within polio eradication. All interviews were conducted in the local or official language, audio-recorded, and transcribed. We employed a deductive coding approach and used four gender analysis domains to explore data at the household, community, workplace, and organizational levels.

**Results:**

We completed 196 interviews globally and within each partner country; 74.5% of respondents were male and 25.5% were female. Male polio workers were not allowed to enter many households in conservative communities which created demand for female vaccinators. This changed the dynamics of front-line program teams and workplaces and empowered many women to enter the workplace for the first time. However, some faced challenges with safety and balancing obligations at home. Women were less likely to receive promotions to managerial or supervisory roles; this was also reflected at the global level. Some described how this lack of diverse management and leadership negatively affected the quality of program planning, delivery and limited accountability.

**Conclusions:**

Gender power relations play an important role in determining the success of global health programs from global to local levels. Without consideration of gender, large-scale programs may fail to meet targets and/or reinforce gender inequities. Global disease programs should incorporate a gender lens in planning and implementation by engaging men and boys, supporting women in the workplace, and increasing diversity and representation among leadership.

## Background

After a century of feminist advocacy and years of discourse on gender inequities driving development, healthcare, and governance, gender equality is recognized as a key determinant of health and wellbeing [1, 2]. Defined as ‘socially constructed roles, behaviors, activities, attributes, and opportunities that a society considers appropriate for men, women, and people with diverse gender identities, and underpinned by power relations’ [3, 4], gender remains a complex notion [5].

Globally, gender as a barrier or facilitator in achieving health outcomes has increasingly been documented, wherein it interacts with social and economic stratifiers such as age, education, ethnicity, religion, disability, etc., influencing individuals’ access to health services, control of resources, and needs and vulnerabilities [6, 7]. Women live longer than men but also suffer longer with chronic diseases [8, 9]. Pregnancy complications and unsafe abortions remain a significant cause of death in many settings, with one third of girls married before they are 18 [10–12]. Gender pay and pension gaps put women at risk of poverty and social exclusion which creates further barriers to accessing health services [13]. And, gendered labor, including increased domestic and reproductive responsibilities and workload, and unpaid labor have adverse effects on a woman’s wellbeing and long-term health [14]. Gender influences not just the risks we take with our health, the risks we face, and whether or not we seek health care, it also impacts how the health system responds to our needs when we are sick or need care [15].

At the same time, the role of gender in global health programming, organization, and financing, while equally important, is frequently overlooked, and the gendered contexts of health systems and decision makers who shape health care delivery are often ignored [15–17]. For example, within global health organizations, gender inequalities continue to define and drive career pathways and opportunities. Women account for up to 75% of the global health workforce and deliver care for over 5 billion people [18], yet only a small fraction of these women hold leadership positions [19, 20], disproportionately representing lower cadres of health workers. Healthcare continues to be delivered by women and led by men [18]. Gendered norms and power relations within global health organizations and programs also result in inequitable access to resources, such as education and training opportunities, distribution of labor and roles, wage and working conditions, social norms and values, and decision making and autonomy [6, 15]. One such global health program which has similarly encountered challenges with gender is the Global Polio Eradication Initiative (GPEI). The GPEI is one of the largest, globally coordinated public health programs in history, spanning across 200 countries for over 30 years. Challenges with gender have included inequitable outcomes for people across gender identities and a failure to prioritize gender equality and equity in organizational practice, including within leadership structures and participation within vaccination activities [5].

Following decades of operations across the globe, the GPEI recognizes that the successes of the global polio eradication efforts are in large part due to the brave and dedicated female health workers, from the front lines, to management, to leadership. In an effort to advance gender equality and gender mainstreaming in programmatic activities as well as organization policies and practices as efforts to eradicate polio continue, the GPEI launched a Gender Strategy 2019–2023 [21]. The strategy aims to promote integration of gendered perspectives into all programmatic aspects; support countries in addressing gender-related barriers to polio vaccination; increase women’s meaningful participation in leadership across levels of the program; and create gender equitable institutional environments.

Similar efforts are ongoing in other global health contexts through initiatives such as the WHO Global Strategy on Human Resources for Health: Workforce 2030, the UN High Level Commission on Health Employment and Economic Growth, Global Health 50/50, Women in Global Health, WomenLift and #LancetWomen. These movements, which focus on advocating for increasing women’s participation in leadership and prioritizing gender in organizational practice, directly impact equitable health care service delivery [17].

The Synthesis and Translation of Research and Innovations from Polio Eradication (STRIPE) project seeks to map, synthesize, and disseminate knowledge and lessons learned from the global polio eradication effort. An explanatory sequential mixed-methods approach [22] was applied to map knowledge at the global level and within 7 consortium countries (Afghanistan, Bangladesh, Democratic Republic of the Congo, Ethiopia, India, Indonesia, and Nigeria). This paper seeks to utilize the qualitative data collected through key informant interviews to describe the role of gender power relations in the polio program at the global, national, and subnational levels, prior to the launch of the GPEI Gender Equality Strategy.

## Methods

### Participants

We conducted key-informant interviews (KIIs) with individuals purposively selected from the ‘polio universe’ (i.e., all individuals who have been directly involved in implementing polio eradication related activities for 12 or more continuous months between 1988 till date), within key global organizations (i.e. the World Health Organization, Rotary International, the US Centers for Disease Control and Prevention, the United Nations Children’s Fund, and the Bill and Melinda Gates Foundation) and within each of the seven consortium countries. The development and application of the polio universe as it pertained to the global organizations and each consortium country is described extensively elsewhere [23]. Respondents were global-, national-, sub-national-, and frontline actors, identified using results from the STRIPE quantitative survey, which explored implementation processes, contextual factors that acted as facilitators or barriers to program implementation, and the level at which they originated [22]. We reviewed survey responses to identify individuals who highlighted representative challenges within each country and at the global level. These individuals were then prioritized to ensure representativeness across key positions, organizations, areas of expertise, geography, and levels within the health system, as well as the ability to speak to core program components. Participants were recruited via email, by the JHU team for global actors and by country teams for actors within their respective countries.

### Data collection

The KII tool was designed to explore nuances of implementation challenges within polio eradication, and strategies for addressing these challenges in addition to their consequences (both intended and unintended). The interview guide focused on questions regarding the organization of the polio program, how it changed over time, internal and external contextual factors, strategies designed and/or tried to overcome barriers, and key lessons learned. Specifically, in exploring implementation challenges, the interview guide included probes to understand if and how the challenges related to gender or gender dynamics, such as differences in access to education, benefits, or other resources, division of labor, and decision-making power. All interviews were conducted in-person or via skype by trained qualitative researchers (2 interviewers at the global level and 3–4 interviewers within each consortium country). Interviewers at the country-level included researchers and research assistants who completed a 2 or 3-day training on the interview guide and best practices in conducting qualitative research. The JHU team developed a training manual which was shared with all country-teams and incorporated into training and the manual covered processes for recruitment, interview recording and transcription, memoing, and data management.

All interviews were conducted in the local or official languages in each country, audio-recorded, transcribed verbatim, and translated into English. Global interviews were conducted and transcribed in English. Data were collected between January and March, 2019.

### Data analysis

We employed a deductive coding approach, deriving codes from two guiding conceptual frameworks, the Consolidated Framework for Implementation Research (CFIR) and the Socio-Ecological Model (SEM). The CFIR is a validated framework widely used in low-and middle-income countries (LMICs), comprised of 5 domains including characteristics of individuals, organizational setting, external setting (e.g. political and social environments), characteristics of the intervention (e.g. the polio program), and process of implementation. This framework was designed to support the development of tools, analysis processes, and synthesis and reporting [24]. CFIR constructs were analyzed across socio-ecological levels drawing from the SEM, which considers complex relationships between factors that influence the individual, interpersonal, organization, community, and larger environment [25]. Combining these two frameworks allowed for the discovery of interactions between different domains for barriers, strategies, and consequences, across levels.

Additionally, a ‘gender’ code was developed to capture instances where gender played a role. All data coded against the ‘gender’ code were reviewed and mapped onto a gender analysis matrix. A gender analysis matrix provides a way to organize information for gender analysis and identifies key gender-related considerations relevant for health and health systems including access to resources; distribution of labor, practices, and roles; norms, values, and beliefs; and decision-making power and autonomy [26].

Coding and subsequent analysis were conducted in Dedoose© a qualitative analysis software. Four reviewers conducted a pilot test on two interviews from two countries, applying the original codebook to both interviews. A meeting was held among the four reviewers to identify any discrepancies in codes and reach consensus on coding approaches moving forward. Each reviewer was then assigned interview transcripts for a set of countries and coded all interviews for those countries. This research was determined “Non-Human Subjects” by the Johns Hopkins Bloomberg School of Public Health Institutional Review Board (IRB). IRB approval was also received by each country institution.

## Results

We conducted 196 interviews across the 7 consortium countries and at the global level. A majority of participants worked at the national level (43.4%) and within government (49%). Among all KII respondents, 74.5% were men and 25.5% were women (Table 1).

**Table 1:** Characteristics of KII Participants

	Men 146 (74.5%)	Women 50 (25.5%)	Total (*N* = 196) N (%)
**Country distribution**			
Global	14	4	18 (9.2%)
Afghanistan	23	5	28 (14.2%)
Bangladesh	14	3	17 (8.7%)
Democratic Republic of Congo	18	6	24 (12.2%)
Ethiopia	26	4	30 (15.3%)
India	20	5	25 (12.8%)
Indonesia	13	13	26 (13.3%)
Nigeria	18	10	28 (14.3%)
**Levels worked**			
Global	14	4	18 (9.2%)
National	64	21	85 (43.4%)
State/District	57	14	71 (36.2%)
Sub-district/Frontline	11	11	22 (11.2%)
**Organizational representation**			
GPEI partners	50	11	61 (31.1%)
Government	71	25	96 (49.0%)
Implementing organizations	16	7	23 (11.7%)
Research/Academic organizations	4	1	5 (2.6%)
Other	5	6	11 (5.6%)

Using four gender analysis domains, we explored the data at the household, community, workplace, and organizational levels using a socio-ecological model. Key findings are presented below:

### Gender at home and in the community

Gender dynamics played an important role in household decision making for determining who, if anyone, was to receive the polio vaccine. Across contexts, male heads of house held decision-making power and women were not viewed as being autonomous.

*They [women] are sure that we are vaccine staff; they don’t open door, we have these kind of refusals, many says that my husband said if you vaccinate the child I will divorce you. We have faced cases like this, we even convinced her father in law and husband but the wife was resisting, [ok, you mean women cannot decide?] yes*. - Afghanistan, M, GPEI official

This had implications for polio program strategies which needed to ensure that husbands were reached in addition to mothers.

This approach was challenging in some settings where vaccination was seen as “women’s stuff” (e.g. DRC) and where women might be viewed as more motivated to learn about vaccines (e.g. Bangladesh).

Young boys were sometimes hidden from polio workers due to a belief that the vaccine might sterilize them. At the global level there was a concern about eliminating the disease if boys were hidden. One respondent reported that in India Muslim boys were, at one point, at four times the risk of getting polio than girls, in part because their families refused them the vaccine.

*They used to tell that give it to the girl child and don’t give it to the boy. The other thing is we are working in a mostly patriarchal society in [India], so if the husband says ‘don’t give it to the child’, then there is no way [the mother] will allow you. -* India, F, National

In other contexts, including within Ethiopia and other regions of India and Afghanistan, boys were sometimes prioritized over girls for the vaccine.

In some settings male polio workers were not allowed to enter households, particularly if the male head of house was not present. This created a great need to recruit and train female workers who could access homes in more conservative communities. In some communities the program provided an opportunity for women to gain employment for the first time, which allowed for their advancement as ‘professionals’ and bread winners.

*We were able to get in contact with so many Muslim communities, all kinds of Muslim communities, all kinds of mosque and Imams and other social leaders of Muslim community. [ … ] The fact that these Muslim girls were allowed to work, and the fact that they all came out, [and] allowed to go house to house. These are huge social changes, I mean you won't find this in any other place and [ … ], we had sparked something new in these young women. That they had the desire to come out and to be recognized, to be seen, you may have heard about many of them, they are like tigresses.* – India, F, GPEI partner

Once trusted by communities, female health workers were able to help other women access additional resources including literacy courses. They also helped raise awareness of other health issues (e.g. hygiene and intimate partner violence).

*Since people trust us, we can help in educating women. We can help in literacy courses for women [literacy is an important issue] this can increase awareness in women. As they say if you teach a man it is one person but if you teach a woman you change a family. We can create awareness through literacy courses for hygiene, violence against women and teach women basic reading and writing. -* Afghanistan, F, Front Line Worker

Within the polio program workforce, gender dynamics at home were also seen as a barrier. Some participants indicated that with more women in the work force the long hours would affect their family life.

*Because the fact during the implementation was more the women who were employed and to occupy them for several hours caused problems in their homes and some returned before the closure of the activities; which delayed the smooth implementation of the implementation* – DRC, M, Sub-national government official.

In India, women who were recruited as community mobilizers were initially paid in cash until the program realized the husbands were collecting the money instead. As a result, the program helped women open individual bank accounts into which the payments were directly deposited. This was viewed as giving women mobilizers more confidence in their work and abilities in addition to independence to pursue work.

Also, while female workers often had more access to conservative households, there remained specific hard-to-reach or unsafe areas where women were not allowed to travel. This included those only traversable by motorbikes which were viewed as inappropriate for women to ride (e.g. Afghanistan) or conflict-affected areas (e.g. Afghanistan and Nigeria). This combination left some communities un-reached and unvaccinated.

### Gender in the workplace

To meet the needs of the program and gain access to conservative communities, women were increasingly hired as front-line workers. This influx of women into the polio program workforce changed the dynamics of program teams and workplaces. All male teams were limited in their scope, creating a need for mixed-gendered teams including both men and women. This maximized access to the communities and in some situations, helped ensure safety of female workers.

*Afghanistan is a conservative society, if we have at least one female in each team for visiting houses this would facilitate the implementation. Two is even better but one is a must because in the families mostly it is mothers staying with children and man cannot access them easily. If a man is in front of a house this is not easy for him but a female volunteer can easily enter the house and family accepts to bring their children. So this is a cultural issue. We also have some gender imbalance and issue in the team so it would be good if we can have female members in the team. This would be good for the team and the program.* – Afghanistan, M, Implementing Partner

*But more importantly operationally speaking is that we've learned that it's extremely important to have females in the vaccination teams and as supervisors. Extremely important. They're much more effective teams. They have much more access inside the homes in many communities where it is inappropriate for men to enter the household, so female members of the vaccination teams are absolutely invaluable, and then in particular having females who are from the same communities*. – Global, M, GPEI partner

While some respondents said that the distribution of labor was not gender-based, others noted that in some settings men were consistently promoted to managerial or supervisory roles while women were hired and kept as front-line workers.

*In the field you had to employ all these young women, and many of the supervisors were men only.* - India, F, GPEI official

*There are lots of such differences that male staff can manage but not female staff. We are not independent and should strictly follow the rules and hierarchy*. – Afghanistan, F, Front-line worker

Some participants described having preferences for working with other men because of the extra cost for ensuring women’s safety (i.e. providing an escort), a perception that men completed jobs faster and better, and a perception that men were more “durable”.

*Yes, at times we’ll preferably want to work with men because they are durable, there are no issues of maybe my baby will cry at home or my husband will come and maybe issues of coming late. -* Nigeria, M, Sub-national, government official

Some female workers also faced harassment from men in the community who would comment on their appearance or refuse to listen to their messages.

### Gender in the organization

When asked about the influencing role of gender within the polio program, many respondents first considered the household and community levels, but upon further thought, some also reflected on how engagement with female workers had brought about changes and opportunities for women more broadly.

*So one of the realizations I think of the polio program over time has just been the important role of women at the community level and making decisions around immunization for their children and the importance of making sure that women are engaged and informed. And one of the really positive knock on effects of the polio program has been just the sheer numbers of local women trained and engaged and again, in Nigeria I hear a lot, "Oh, we love doing this, what's next for us?" And I really would hope and wish that for public health engagement those same women still get to be engaged somehow, I know that the next program or initiative or government system won't have the same money as the polio program does but that's really cool, it's a very empowering at the community level for all those women. –* Global, F, GPEI partner

Many respondents, particularly those at the global level, reflected on the role of gender within the larger polio program’s organization. There was some agreement that men tend to hold more leadership positions overall, though this slowly changed over time. One participant at the global level indicated that there are more men in leadership positions because men have had more opportunities growing up.

*I didn’t necessarily see [program gender policies] translate into potentially greater gender balance in leadership, you know, within WHO and UNICEF, although I think it’s improved over the years, but, I would say that most of my African colleagues in Central Africa, whether they be at the ministerial level or at WHO or UNICEF, I mean, the vast majority of them were men. I think it’s more historical structural, if you will, and opportunities that men had to be able to first, you know, go to school, second, become a doctor, third, to become a, you know, a district health manager, which was typical route that these folks took, and then worked. There were just less women who were educated enough to go to medical school and become doctors and so, I mean, it was always great to have more female staff in leadership positions, but it was a lot less frequent, that’s for sure.-* Global, M, Implementing partner

Another participant further reflected that the program has increasingly emphasized the need for female frontline workers to address barriers to household and community access, but there has not been a similar push for females in leadership positions.

*There are gender dynamics and there is a gender imbalance within the global polio program. It's one of the most male-dominated global programs I’ve ever seen. I don't like it. And I think the program would be better if we had a healthier gender balance, let me put it that way. I think there's been a lot of emphasis put on the importance of having more female front-line workers; that's fine but that is inadequate. We need to have women at all levels of the program including the management in the GPEI management structure. –* Global, M, GPEI partner

The same participant reflected on the implications of this gender imbalance within the polio program including on program design and quality.

*I go to these meetings and I find it personally unacceptable and actually, embarrassing. But I also think that the quality of the program and insights would be better informed if we had more women contributing at various levels and not just on the front line. –* Global*,* M, GPEI partner

One respondent expressed concern that a lack of female supervision also meant a lack of accountability within the program. If male workers could not enter houses, there was no way for male-led teams to follow-up and check on whether children had been vaccinated.

*This relates to the challengeson the ground in every country, corruption and accountability are such a problem, and that's the reason we haven't eradicated in some many places or haven't interrupted transmission. I started working in [a polio endemic country] and at that point there were huge numbers of teams that didn't have a female supervisor which meant that the teams couldn't go in the house and actually check, they had no idea whether the kids were vaccinated. And the gender dynamics of how the polio eradication effort functioned in a society that doesn't allow strange men to enter into a home; it kind of boggles my mind that they went for so long without realizing that was a need. –* Global, F, GPEI partner

In addition to gender, some participants noted the importance of racial diversity and indigeneity among organizational leadership. One interviewee at the global level criticized the GPEI and suggested an intersectional approach for future global initiatives.

*And so I would say that kind of race and ethnic diversity too is like gender in the whole GPEI effort, it's a really fraught conversation and on one hand it's a really awesome coming together of a lot of different cultures and viewpoints at its best, at its worst, it's kind of a very neo-colonial approach to getting things done. Again, that’s one of the criteria I would add to the global community’s list of, “How would we consider shaping another eradication initiative and are we taking these things into account?” –* Global, F, GPEI partner

## Discussion

Evidence from this study shows the important role that gender power relations play in determining the success of global health programs. Without consideration of gender, global health programs, like the polio eradication program in this study, may fail to meet their required targets, or the targets they do meet are likely to reinforce gender inequity. Below we reflect on some of the key gender considerations that emerged from the study and how these should be incorporated within the polio eradication program going forward, as well as other global health programs.

### Gender at home and in the community

It is important that implementers consider who holds decision-making power within the household, and whether their lack of involvement may affect who receives vaccination or who does not. Evidence shows that in families where women hold decision-making power, including in relation to health, a greater proportion of resources is devoted to children [27]. The reality is, however, that in many LMICs, men continue to hold decision-making power within households, deciding who receives care and when. Male engagement within global health programs which are targeted towards women and children therefore becomes very important [28, 29]. Equally important is how men are engaged if programs are to avoid perpetuating unequal gender norms, roles, and relations. A useful resource for global health program implementers is the Interagency Gender Working Group’s Do’s and Don’ts for engaging men and boys [30]. Appropriately engaging men in polio vaccination programs can help to dispel the belief that vaccination is a women’s issue and ensure that boys and girls are both vaccinated.

### Gender in the workplace

The gendered contexts in which polio vaccination house campaigns and social mobilization strategies are implemented, which affects who is and is not allowed to enter homes or engage with women and girls, exemplifies the need to recruit and train female workers. On one hand women are not able to participate equally and meaningfully in vaccination activities due to limited access to services and control (i.e. decision making) over resources, restricted social and physical mobility, and in some places the lack of a safe and respectful work environment. On the other hand, female vaccinators are preferred by communities and have easier access to households – a key strategy in reaching every last child. Increasing the representation of female workers within polio vaccination programs not only helps to increase coverage of vaccination [31], but also supports the empowerment of women in communities in which employment is limited [32], and can improve health outcomes [33]. Increasing women’s involvement in the workforce, however, can have unintended consequences within the household. Engaging male partners when women are being recruited and trained can help to increase their support for women’s employment and ease any tensions that may result as a result of women’s involvement in the workforce.

### Gender in the organization

The gender segregated nature of the polio vaccination workforce is reflected in the lack of representation of women within this study. Some STRIPE consortium partners indicated that it was difficult to identify women participants, other than front-line workers, in part because there were fewer available in each setting’s polio universe. The polio program has historically maintained a traditional gender structure with primarily male leadership. While it is important to emphasize increasing the number of female front-line workers, it is equally important to increase women’s representation within leadership. Lack of diversity within leadership and decision-making structures affects what is prioritized within global health programs. Evidence from other sectors shows that a lack of diversity within leadership and a failure to leverage women’s talent and expertise limits the effectiveness of response efforts [34]. Increasing diversity within leadership structures helps to ensure that diverse perspectives and needs are incorporated, limiting the traditional groupthink that occurs in many global health programs, and increasing overall accountability [34]. Increasing diversity does not only mean increasing the representation of women within decision-making, but the representation of other marginalized and vulnerable groups, such as racial or ethnic minorities. It is therefore important that implementers consider how gender intersects with other social stratifiers, such as race, ethnicity, income, education, or disability – both among those involved in implementation and delivery and beneficiaries of programs.

### Gender strategy

The GPEI Gender Equality Strategy 2019–2023 [21] emphasizes the importance of engaging women at all levels of the GPEI, stating that increased participation of women is integral to reaching every last child with a vaccine. Further, the Gender Equality Strategy acknowledges that understanding the impact of gender on development, as well as health and emergency outcomes, is critical to polio eradication. The key findings of the STRIPE gender analysis parallel the four objectives of the Strategy: 1) Integration of a gender perspective into programming and organizational structures, 2) Addressing gender-related barriers to increase polio vaccination coverage, 3) Increasing meaningful participation of women at all levels of polio eradication, and 4) Creating gender-equitable culture and environments at the institutional level. These objectives reflect the need to address gender across SEM levels, supported by the evidence from this study [21].

In addition to providing objectives, the strategy outlines a strategic framework and provides the WHO gender-responsive assessment scale. Implementing organizations can utilize these tools to assess current gender-responses and to develop a plan reach the goal of “Increased number of girls and boys reached with polio vaccines to support the achievement of a polio-free world.” Seven areas of focus, along with actions from the GPEI are outlined. If implemented effectively, the GPEI will move all programming to the ‘gender-sensitive’ level of the WHO gender-responsive assessment scale.

Implementation of the Strategy has potential to sustainably impact efficacy of the GPEI, across SEM levels. First, the proposed changes will improve the data available to understand gender differences, through the use of sex-disaggregated data and gender-sensitive indicators. At the organizational level, the GPEI has set gender parity goals, which can help to increase training and skills needed to integrate gender into all aspects of GPEI work, and prevent sexual exploitation, abuse, and harassment [21]. Given the breadth of the polio initiative, successful implementation of the GPEI Gender Equality Strategy 2019–2023 has the potential to improve gender equality across current and future global health initiatives.

### Strengths and limitations

This study uniquely captures the experiences of participants at the global, national, subnational, and front-line levels across 7 countries. Interview tools were designed to illicit responses specific to gender ensuring participants had the opportunity to reflect on this issue. However, given each country’s polio universe, it was difficult to create parity among the number of female and male interviewees across country settings. Data collectors in different settings received varying levels of training and may have addressed the gender-related questions differently though each country team was encouraged to recruit a diverse cadre of data collectors to ensure female data collectors were available, specifically for interviewing front line health workers. Finally, we noted that many of the interviews were not conducted in English but were translated and analyzed in English. Nuances in the original language may have been lost and could have influenced our analysis.

## Conclusions

For global programs to meet their goals and improve health, gender must be addressed at every level, from the household and community to management and organizational leadership. Country specific findings can be used to begin national dialogues on the role of women leaders in public health dialogues on the role of women leaders in public health. This study reinforces the need for gender equality strategies in global health and provides recommendations including engaging men and boys, supporting women in the workplace, and creating diverse organizations.

## Data Availability

Data is available upon request.

## Abbreviations

CFIR: Consolidated Framework for Implementation Research
GPEI: Global Polio Eradication Initiative
LMIC: Low-and middle-income country
KII: Key-informant interview
SEM: Socio-ecological model
STRIPE: Synthesis and Translation of Research and Innovation in Polio Eradication
